# The clinical potential of PDL-1 pathway and some related micro-RNAs as promising diagnostic markers for breast cancer

**DOI:** 10.1186/s10020-025-01137-1

**Published:** 2025-03-19

**Authors:** Eman A. Al-Sharabass, Motawa E. EL-Houseini, Heba Effat, Sherif Abdelaziz Ibrahim, Mona S. Abdellateif

**Affiliations:** 1 Zoology Department, Faculty of Science, Cairo University, Cairo, Egypt; 2 Medical Biochemistry and Molecular Biology, Cancer Biology Department, National Cancer Institute, Cairo University, Cairo, Egypt

**Keywords:** Breast cancer, MIC-B, MiR-155, MiR-195, PDL-1, FOXP3, And CTLA-4

## Abstract

**Background:**

Immune checkpoint pathways play important roles in breast cancer (BC) pathogenesis and therapy.

**Methods:**

Expression levels of programmed cell death protein 1 (PD-1), cytotoxic T-lymphocyte–associated antigen 4 (CTLA-4), programmed death-ligand 1 (PD-L1), Forkhead box P3 (FOXP3), miR-155, and miR-195 were assessed in the peripheral blood of 90 BC patients compared to 30 healthy controls using quantitative real-time PCR (qRt-PCR). The plasma level of soluble MHC class I chain related-protein B (MIC-B) protein was assessed using the enzyme linked immunosorbent assay (ELISA) technique. The data were correlated to the clinico-pathological characteristics of the patients.

**Results:**

There was a significant increase in the expression levels of PDL-1 [17.59 (3.24–123), p < 0.001], CTLA-4 [23.34 (1.3–1267), p = 0.006], PD-1 [10.25 (1–280), p **< **0.001], FOXP3 [11.5 (1–234.8), p = 0.001], miR-155 [87.3 (1.5–910), p < 0.001] in BC patients compared to normal controls. The miR-195 was significantly downregulated in BC patients [0.23 (0–0.98, p < 0.001]. The plasma level of MIC-B was significantly increased in the BC patients [0.941 (0.204–6.38) ng/ml], compared to the control group [0.351 (0.211–0.884) ng/mL, p < 0.00].

PDL-1, CTLA-4, PD-1, and FOXP3 achieved a specificity of 100% for distinguishing BC patients, at a sensitivity of 93.3%, 82.2%, 62.2%, and 71.1% respectively. The combined expression of PDL-1 and CTLA-4 scored a 100% sensitivity and 100% specificity for diagnosing BC (p < 0.001). The sensitivity, specificity, and AUC of miR-155 were 88.9%, 96.7%, and 0.934; respectively (p < 0.001). While those of miR-195 were 73.3%, 60%, and 0.716; respectively (p = 0.001). MIC-B expression showed a 77.8% sensitivity, 80% specificity, and 0.811 AUC at a cutoff of 1.17 ng/ml (p < 0.001). Combined expression of miR-155 and miR-195 achieved a sensitivity of 91.1%, a specificity of 96.7%, and AUC of 0.926 (p < 0.001). Multivariate analysis showed that PDL-1 (OR:13.825, p = 0.004), CTLA-4 (OR: 20.958, p = 0.010), PD-1(OR:10.550, p = 0.044), MIC-B (OR: 17.89, p = 0.003), miR-155 (OR: 211.356, P < 0.001), and miR-195(OR:0.006, P < 0.001) were considered as independent risk factors for BC.

**Conclusions:**

The PB levels of PDL-1, CTLA-4, PD-1, FOXP3, MIC-B, miR-155, and miR-195 could be used as promising diagnostic markers for BC patients.

## Background

Breast cancer (BC) is a heterogenous group of diseases with variable molecular and biological characterizations (Hou et al. 2022). It is the most common malignancy in females and remains the second leading cause of cancer-related death in women worldwide, that represented 15% of all female deaths in 2022 (Siegel et al. 2023). Several prognostic and predictive biomarkers emerged in the last few years that guided personalized therapy for BC patients (Freelander et al. 2021). Therefore, the 8th edition of the American Joint Committee on Cancer (AJCC) Staging established combined multiple molecular markers in addition to the traditional clinical staging, estrogen receptors (ER), progesterone receptors (PR), and Herceptin (HER-2) biological markers for the evaluation of BC (Giuliano et al. 2018).

The immune system regulation plays a central role in the development and progression of BC (Kipkeeva et al. 2022). One of the main components of the immune system is the checkpoint molecules that proved fundamental functions in cancer development as well as cancer immunotherapy (Rakha et al. 2022). The checkpoint molecules comprise many factors such as the programmed death-ligand 1 (PD-L1), programmed cell death protein 1 (PD-1), and the cytotoxic T-lymphocyte–associated antigen 4 (CTLA-4). PD-1 and CTLA-4 are co-inhibitory receptors expressed on the surface of T-lymphocytes, where they bind to their ligand (PDL-1 and CD80/CD86 ligands; respectively) expressed on the surface of regulatory immune cells and some types of cancers (Beckers et al. 2016; Cimino-Mathews et al. 2016). The binding of these PD-1 and CTLA-4 receptors with their ligands results in the inhibition of T-cells activity and suppression of the antitumor immune response (Li et al. 2016). Therefore, the use of immune checkpoint inhibitors (ICIs) achieved success in many clinical trials involving melanomas, renal cell carcinoma, non-small cell lung cancer, head and neck squamous cell carcinomas (Kipkeeva et al. 2022; Seidel et al. 2018). According to these studies, the use of PD-1 inhibitor (nivolumab) together with CTLA-4 inhibitor (ipilimumab) were approved by the FDA for the treatment of different types of metastatic tumors (Kooshkaki et al. 2020). Additionally, the FDA approved SP142 and 22C3 (PDL-1 assays) as two diagnostic tests to select patients eligible for atezolizumab and pembrolizumab checkpoint inhibitors treatment (Nanda et al. 2016; Adams, et al. 2019; Schmid et al. 2020).

The Forkhead box P3 (FOXP3) is transcription factor having a DNA-binding domain that inhibits the expression of the target gene (Tanaka and Sakaguchi 2019). The expression of FOXP3 is mostly specific for the T-regulatory cells (T-regs), which is an important regulator of the immune function (Wing et al. 2019; El-Houseini et al. 2022). Several studies investigated the prognostic role of FOXP3 in BC, however the data is still conflicting (Shou et al. 2016). Some studies reported the poor prognostic role of FOXP3 in BC patients, while other studies reported a paradoxical role (Sun et al. 2014; Maeda et al. 2014; Mahmoud et al. 2011).

Micro-RNAs (miRNAs) have a pivotal role in several vital processes in the human body including metabolism, cancer pathogenesis, and immune regulation (Zhang et al. 2019). The miRNAs are small non-coding RNA that control gene expression through post-transcriptional regulation (Kipkeeva et al. 2022). Different miRNAs had been reported to regulate BC pathogenesis, immune cells’ functions, IC gene expression, and accordingly anti-tumor immune response (Shao et al. 2021; Xu et al. 2020; Soleimani et al. 2021). One of these miRNAs is the miR-155 that is known as a common oncogenic microRNA present on the chromosome 21q21.3 in the B cell integration cluster region (Xu et al. 2022). It was found to be upregulated in several cancers including leukemia, breast, thyroid, pancreatic, and lung cancers (Hassan et al. 2020; Iorio et al. 2005; Nikiforova et al. 2008; Yanaihara et al. 2006; Greither et al. 2010). miR-155 has a role in cancer pathogenesis by regulating the JAK-STAT, MAPK, TGF-β, and FOXO3a signaling pathways (Kipkeeva et al. 2022; Seto et al. 2018). It was also found to regulate dendritic cell function and IL-12 secretion through targeting p27kip1, KPC1, and SOCS-1 signaling pathways (Lu et al. 2011).

Another miRNA, a tumor suppressor miR-195, was reported to have an important function in suppressing cell proliferation and cancer progression (McAnena et al. 2019). It is a member of the miR-15 family, and its gene is present in the 17p13.1 chromosome (Yu et al. 2018). Several studies investigated the diagnostic and prognostic value of miR-195 in BC, however its precise role is still unclear. Some studies found an increased level of miR-195 in BC patients (Fan et al. 2018; Zhang et al. 2016), however others reported its decreased level in BC patients in comparison to the control individuals (McAnena et al. 2019; Luo et al. 2014).

The MHC class I chain related-protein B (MIC-B) is a member of (MIC) gene family, which is located at the HLA region (Bahram and Spies 1996). The MICA and MIC-B are membrane-bound molecules present on the surface of the tumor cells, where they bind to the natural killer group 2D (NKG2D) on NKs. This binding results in the activation of γδ T cells and NK cells mediated antitumor immune response (Bauer et al. 1999). On the other hand, the soluble isoform of MIC-A/B (sMIC-A/B), which is produced by the shedding of the membrane-bound molecule MIC-A/MIC-B in the plasma (Waldhauer et al. 2008). The MIC-A/B and sMIC-A/B had been investigated in several research studies, where conflicting data regarding the clinical value of these molecules were reported (Zhao et al. 2017). Some series concluded that MIC-A/B were good prognostic markers in melanoma, pancreatic, hepatocellular, lung, and colorectal cancers (Fang et al. 2014; Duan et al. 2011; Okita et al. 2016; Watson et al. 2006). While others proposed the unfavorable prognostic role of MIC-A/B in ovarian, breast, and non-small cell lung cancers (Madjd et al. 2007).

The present study aimed at investigating the clinical relevance of the PDL-1 pathway including PDL-1, CTLA-4, and PD-1, as well as FOXP3, miR-195, miR-155, and MIC-B expression levels in the diagnosis of BC. In addition, correlating the expression levels of those markers with the clinic-pathological features of the patients. This might help for enhanced detection and early diagnosis of patients suffering from BC. Also, it could provide a target for a potential personalized therapy.

## Methods

The is a case control study included 90 females confirmed for BC diagnosis at the national cancer institute (NCI), Cairo university, compared to 30 normal age and sex matched healthy individuals. The study was performed at the NCI during the period from February 2023 to January 2024. The included patients were subjected to complete history taking, full clinical examination, laboratory, and radiological assessment. The expression levels of PDL-1, CTLA-4, PD-1, FOXP3, miR-155, and miR-195, as well as the MIC-B protein were investigated in the PB of newly diagnosed BC patients prior to receiving any treatment that might influence gene expression levels.

### Sample collection

Peripheral blood samples of 5mL were drawn from the participated patients and control individuals in a sterile ethylenediamine tetraacetic acid (EDTA) coated tubes. The tubes were transferred to the cell culture lab for the isolation of the peripheral mononuclear cells (PMNCs).

### Isolation of PMNCs

The PMNCs were isolated using Ficoll-Hypaque density gradient centrifugation (1.077 g/mL, Serana Europe GmbH). The separated PMNCs were centrifuged at 1200 xg and washed with 2 mL of phosphate buffer saline (PBS). Finally, the cells were lysed in the QlAzol® Lysis Reagent (Qiagen, Germany) for total RNA isolation.

### Extraction of the total RNA

The total RNA was extracted using miRNeasy Mini Kit (cat. no. 217004, Qiagen, Germany), according to the manufacturer’s instructions. The concentration and the purity of the extracted RNA was measured by a NanoDrop-2000 spectrophotometer (ThermoFisher Scientifc, USA).

Complementary (cDNA) was transcribed from 8 µl mRNA using EasyScript® First-Strand cDNA Synthesis SuperMix Kit (Trans, Cat.no. AE301-02, China), according to the Kit guidelines. Also, 4 µl of the template RNA (5 ng/μl) was used for reverse transcription of the miRNA using miRCURY LNA reverse transcription kit [Qiagen, Germany] following the manufacturer’s instructions.

### Quantitative real‑time PCR [RT‑qPCR]

The mRNA and miRNA expression were quantified using Solg™2X Real-Time PCR Smart mix (Including SYBR® Green I, Cat. No. SRH83 M40h, Korea), and the miRCURY LNA SYBR® Green PCR Kit (cat. nos. 339345, Qiagen, Germany), respectively. The Primer assays for the PDL-1, CTLA-4, PD-1, FOXP3, miR-155, and miR-195 were obtained from Qiagen, Germany (Table 1). β -Actin and RNU1A1 were used as endogenous controls for normalization of the assessed mRNAs and miRNAs, respectively. The amplification reactions were performed in triplicate using the ViiA™ 7 PCR system [Applied Biosystems, USA] according to the manufactures’ instructions. The relative expression of the assessed genes and miRNAs were assessed by the comparative method using the Equation 2^−ΔΔCt^ (Livak and Schmittgen 2001).

**Table 1:** Clinical characteristics of the breast cancer patients

Patients’ characteristics	Frequency (%)
Sex	
Female	90 (100)
Tumor type	
IDC	90 (100)
laterality	
Rt	56 (62.2)
Lt	34 (37.8)
Tumor grade	
1	2 (2.2)
2	70 (77.8)
3	16 (17.8)
4	2 (2.2)
ER	
Negative	18 (20.0)
Positive	72 (80.0)
PR	
Negative	20 (22.2)
Positive	70 (77.8)
Her	
Negative	68 (75.6)
Positive	22 (24.4)
LNs	
Negative	16 (17.8)
Positive	74 (82.2)
Metastasis	
Negative	78 (86.7)
Positive	12 (13.3%)

### Assessment of soluble MIC-B protein level

The protein levels of the MIC-B were assessed in the plasma of the BC patients and the control group. This was performed using the enzyme linked immunosorbent assay (ELISA) MIC-B assay kit (Cat.no. SEA870Hu, cloud-clone. Corp) according to the manufacture guidelines.

### Statistical analysis

Data analyses were analyzed using IBM SPSS Statistics Version 22. Quantitative data were expressed as median and range according to the performed normality test. Qualitative data were presented as frequency and percentage. Comparison between categorical variables were done using chi-square test or fissure exact as appropriate, while comparison between numerical variables was performed using the Mann–Whitney test and Kruskal-Walli’s test. The Area under the receiver operating curve (ROC) was done to assess the diagnostic value of the tested markers in the BC patients. The cut-off value was determined as the value that achieved the maximal sensitivity and the maximal specificity for the tested marker (the point on the ROC curve the has the minimum distance to the upper left corner where sensitivity = 1 and specificity = 1). Correlations between the assessed markers were performed using the Spearman correlation coefficient. Univariate and multivariate logistic regression analysis were done to detect independent risk factors for BC. All tests were two-tailed, and the significant level was considered at p-value < 0.05.

## Results

The present study included 90 BC females with invasive ductal carcinoma (IDC) subtype. Most of the patients were grade 2 [70/90 (77.8%)], followed by grade 3 [16/90 (17.8%)]. There were 72/90 (80.0%) patients had positive estrogen receptors (ER) expression, 70/90 (77.8%) with positive progesterone receptor (PR) expression, and 22/90 (24.4%) expressed human epidermal growth factor receptor-2 (Her-2). Lymph node metastasis was detected in 74/90 (82.2%) patients, while distant metastasis was found in 12/90 (13.3%) patients **(**Table 2**)**.

**Table 2:** Association among PDL1, CTLA4, FOXP3, PD1 expression and the clinicopathological features of the patients

		PDL1	P value	CTLA4	P value	PD1	P value	FOXP3	P value
Laterality	Rt	18.5 (3.2–123)	0.960	21.9 (1.3–454)	0.724	12.3 (1–171)	0.215	12.9 (2.2–230)	0.244
	Lt	17.1 (8.2–98.2)		24 (2.3–624)		9.6 (1.2–280)		11.2 (1–197.6)	
Tumor grade	1	NA	0.491	NA	0.513	NA	0.260	NA	**0.005**
	2	17.6 (3–123)		24.3 (1.6–624)		11 (1–280)		6.9 (3–73)	
	3	17 (4–109)		8.9 (1.3–267)		5.4 (2.6–188)		16 (1–230)	
	4	NA		NA		NA		NA	
ER	-ve	19.1 (15–34.7)	0.705	23.6 (1.3–156)	0.896	16.2 (5–171)	0.082	13 (3–230)	0.569
	+ ve	17.6 (3–123)		19.5 (1.6–624)		9 (1–280)		11.5 (1–225)	
PR	-ve	20.7 (8–34.7)	0.812	22 (1.3–156)	0.749	10.7 (5–171)	0.212	12 (3–230)	0.385
	+ ve	17 (3–123)		23.6 (1.6–624)		9.9 (1–280)		11.5 (1–225)	
Her	-ve	17.6 (3–123)	0.725	16 (1.3–624)	0517	8.5 (1–280)	0.220	12 (1–225)	0.564
	+ ve	18 (14–54)		24.5 (1.8–111.8)		15 (1.3–171)		11 (3.9–230)	
LNs	-ve	20.6 (13–108)	0.642	7 (1.3–270)	0.159	9.6 (1.2–86)	0.950	28.6 (1–225)	0.254
	+ ve	17.6 (3.2–123)		24 (1.6–624)		10.6 (1–280)		11 (1.9–230)	
Metastasis	-ve	17.6 (3–123)	0.240	18.4 (1.3–624)	0.849	10.7 (1–280)	0.433	12.5 (1–230)	0.129
	+ ve	35.3 (8–109)		26 (2.8–41)		6.4 (1.3–168)		6.8 (3.9–100)	

Bold variable indicates significant values

### The expression levels of PDL-1, CTLA-4, PD-1, and FOXP3 in BC patients

**Fig. 1:**
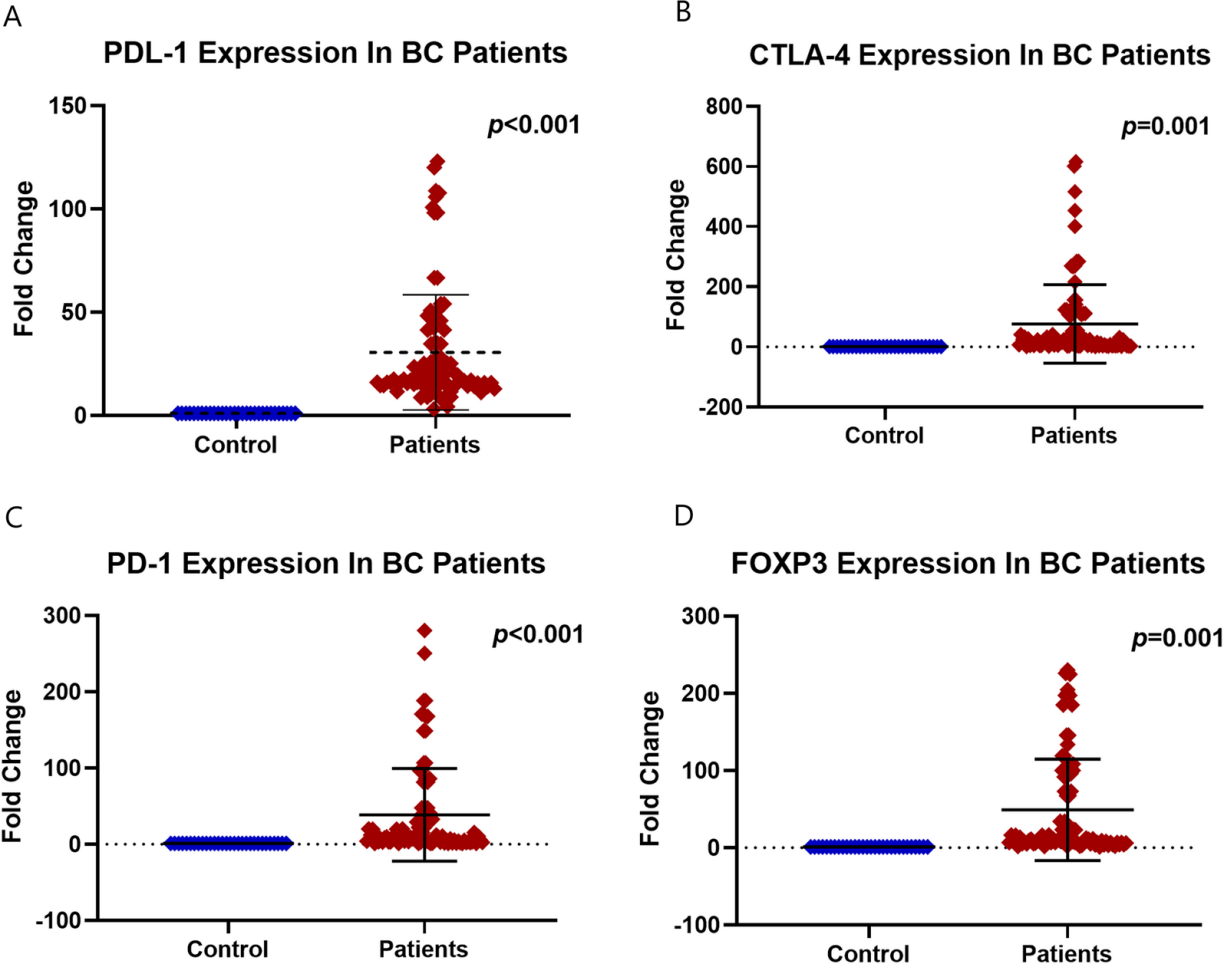
Peripheral blood expression of **A** PDL-1, **B** CTLA-4, **C** PD-1, and **D** FOXP-3 in breast cancer patients compared to normal control group

There was a significant increase in the levels of the assessed immunological markers in the PB of the BC patients compared to the normal control group **(**Fig. 1A-D**)**. These assessed markers included the PDL-1 [the fold change (FC): is 17.59 (range: 3.24–123), p < 0.001], CTLA-4 [FC: 23.34 (range: 1.3–1267), p = 0.006], PD-1 [FC: 10.25 (range: 1–280), p < 0.001**]**, and FOXP3 [FC: 11.5 (range: 1–234.8), p = 0.001**]**.

### The expression levels of miR-155 and miR-195 in BC patients

**Fig. 2:**
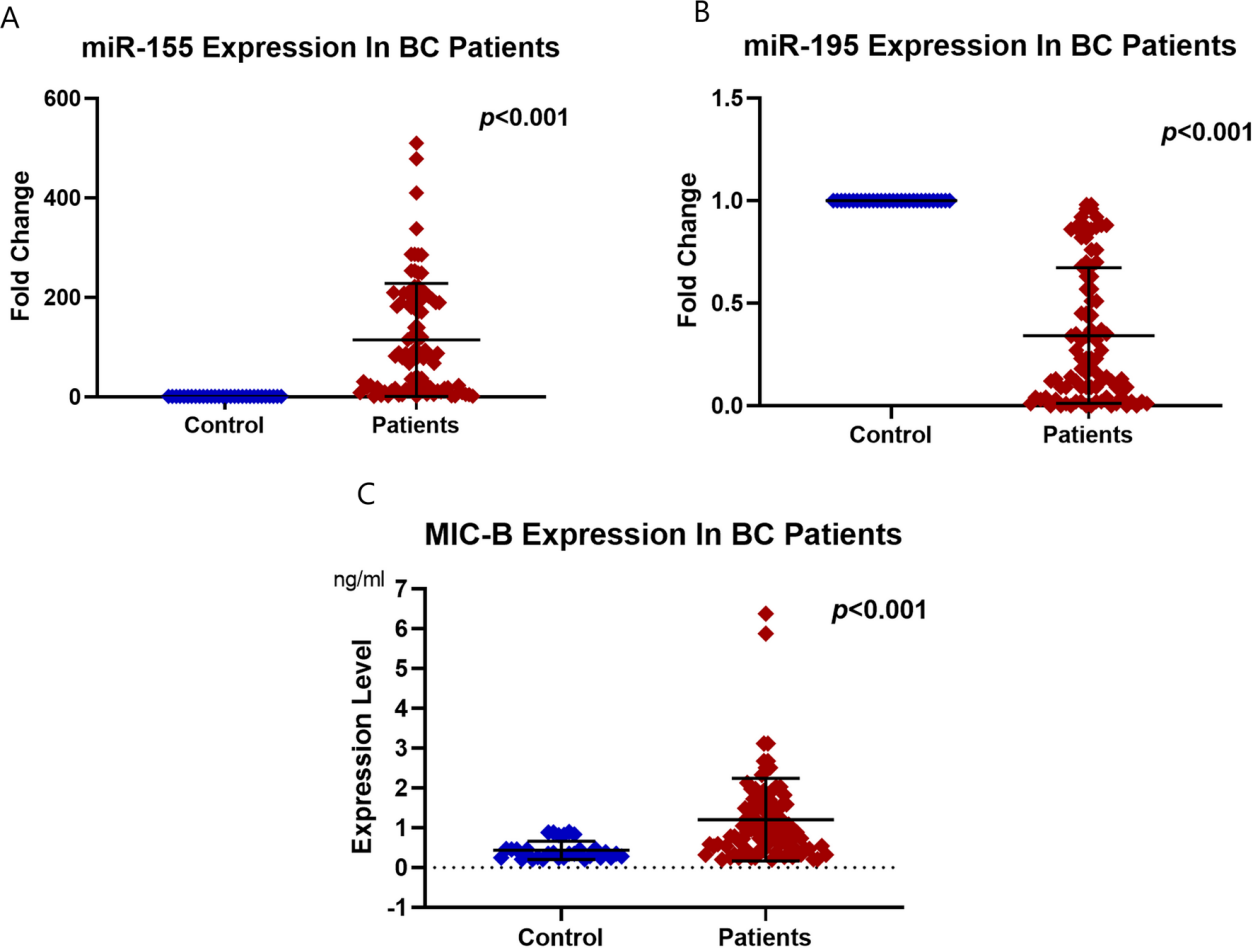
Expression levels of **A** miR-155, **B** miR-195, and **C** MIC-B in the peripheral blood of breast cancer patients compared to normal controls

The expression level of miR-155 was significantly increased in the PB of BC patients compared to the healthy control group [FC: 87.3 (range: 1.5–910), p < 0.001**, **Fig. 2A]. While the expression level of miR-195 was significantly downregulated in the PB of BC patients in comparison to the healthy control group [FC: 0.23 (range: 0–0.98, **p < 0.001, **Fig. 2B].

### Assessment of the plasma protein level of MIC-B in BC patients

The plasma protein level of MIC-B showed a significant increase in the BC patients [0.941 (range: 0.204–6.38) ng/ml], in comparison to the normal control females [0.351 (range: 0.211–0.884) ng/ml, p < 0.001**, **Fig. 2C].

### PDL1, CTLA4, FOXP3, PD1 expression levels in relation to the clinicopathological features of the patients

The expression level of FOXP3 increased significantly in BC patients who had tumor grade 3 [FC: 16 (range: 1–230) compared to those with grade 2 [FC: 6.9 (range: 3–73), p = 0.005]. However, there was no significant association detected between the clinic-pathological features of the assessed patients and the expression levels of PDL1, CTLA4, and PD1 (p > 0.05 for all, Table 2).

### miR-155, miR195, MIC-B expression levels in relation to the clinicopathological features of the patients

The expression level of miR-155 increased significantly in patients with distant tumor metastasis [FC: 187 (range: 31–286)], compared to those negative for distant metastasis [FC: 78 (range: 1.5–210), p < 0.001].

Regarding the miR-195 expression, it was significantly increased in patients with right side BC [FC: 0.36 (range: 0–0.92), in comparison to those with left side BC [FC: 0.12 (range: 0–0.98), p = 0.018]. The miR-195 was significantly downregulated in patients with positive expression of ER [FC: 0.12 (range: 0–0.96)], relative to those with negative ER on the tumor cells [FC: 0.76 (range: 0.14–0.98), p < 0.001]. Similarly, it was significantly decreased in patients with positive PR [FC: 0.16 (range: 0–0.96)], compared to those with negative PR [FC: 0.73 (range: 0.04–0.98), p = 0.009]. On the other hand, there was no significant association between MIC-B expression and any relevant clinic-pathological features of the assessed patients (p > 0.05 for all). Other data were shown in Table 3.

**Table 3:** Association among miR-155, miR195, MIC-B expression and the clinicopathological features of the patients

		miR-155	P value	miR-195	P value	MIC-B	P value
Laterality	Rt	91.7 (1.5–479)	0.057	0.36 (0–0.92)	**0.018**	0.95 (0.20–6.4)	0.803
	Lt	30.8 (1.5–510)		0.12 (0–0.98)		0.89 (0.25–2.68)	
Tumor grade	1	NA	0.637	NA	0.826	NA	0.172
	2	87.3 (1.5–479)		0.23 (0–0.98)		0.89 (0.25–6.68)	
	3	88.3 (2–510)		0.26 (0.01–0.87)		0.84 (0.32–1.6)	
	4	NA		NA		NA	
ER	-ve	151 (1.5–338)	0.133	0.76 (0.14–0.98)	**p < 0.001**	1.08 (0.20–2.13)	0.872
	+ ve	82 (1.5–510)		0.12 (0–0.96)		0.92 (0.20–6.38)	
PR	-ve	120 (0.5–338)	0.308	0.73 (0.04–0.98)	**0.009**	0.99 (0.21–2.13)	0.509
	+ ve	84.7 (1.5–510)		0.16 (0–0.96)		0.94(0.20–6.38)	
Her	-ve	87.3 (1.5–510)	0.481	0.2 (0–0.92)	0.244	0.92 (0.20–6.38)	0.599
	+ ve	99 (2–338)		0.23 (0–0.98)		1.1 (0.21–2.13)	
LNs	-ve	58.2 (4.6–479)	0.899	0.19 (0.02–0.76)	0.966	0.72 (0.38–1.83)	0.526
	+ ve	88.3 (1.5–510)		0.23 (0–0.98)		0.97 (0.24–6.13)	
Metastasis	-ve	78 (1.5–210)	**p < 0.001**	0.23 (0–0.98)	0.887	0.89 (0.24–5.13)	0.669
	+ ve	187 (31–286)		0.17 (0.02–0.96)		0.86 (0.25–1.98)	

Bold variables indicate significant values

### Diagnostic significance of the assessed markers for breast cancer

**Fig. 3:**
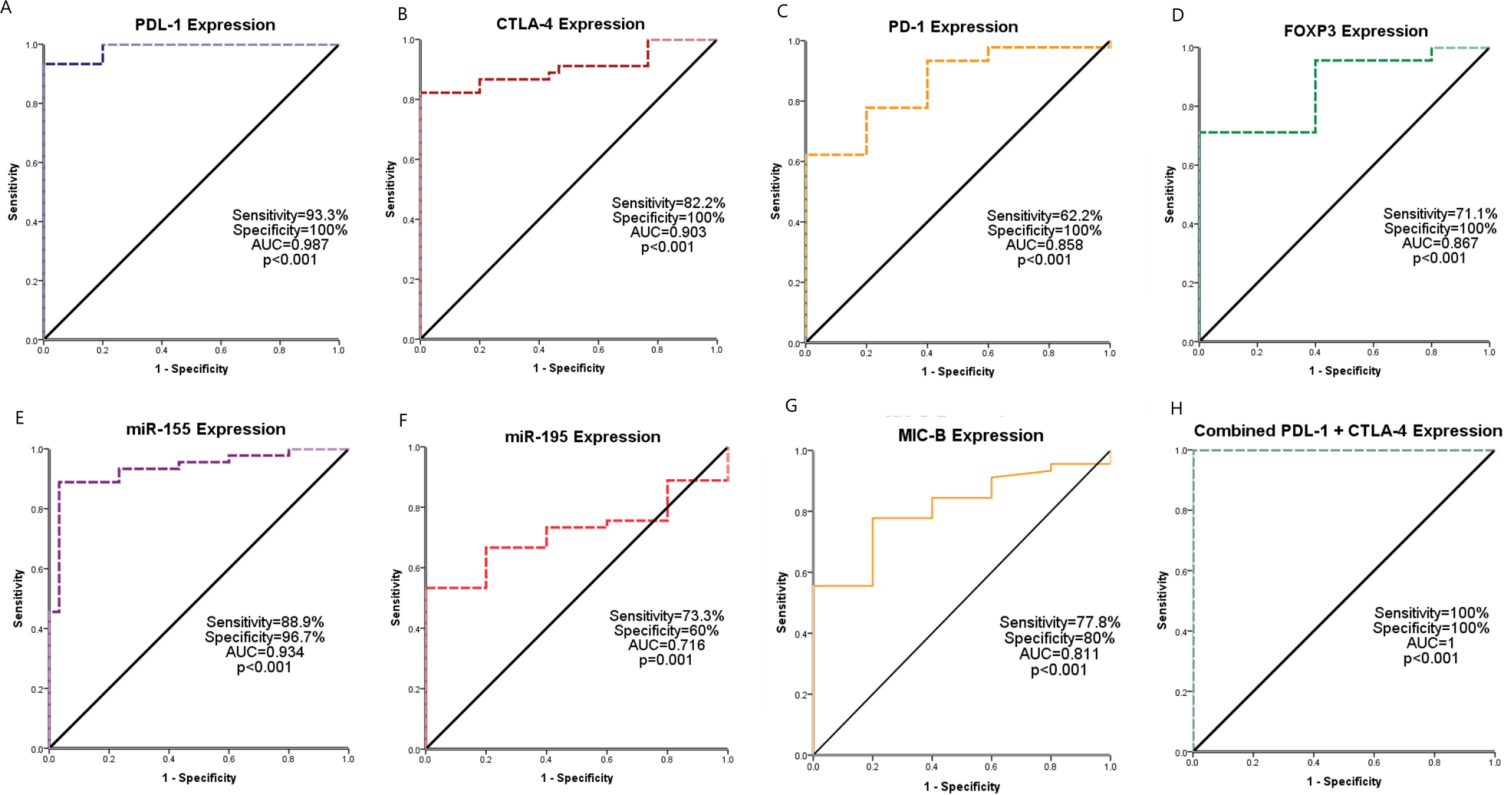
ROC curve analysis of **A** PDL-1, **B** CTLA-4, **C** PD-1, **D** FOXP-3, **E** miR-155, **F** miR-195, **G** MIC-B, and **H** combined PDL-1 and CTLA4 for the diagnosis of breast cancer against normal control

The ROC curve analysis revealed that the sensitivity, specificity, and area under curve (AUC) for PDL-1 were 93.3%, 100%, and 0.987; respectively, at a cutoff value of 1.29 (p < 0.001), and those for CTLA-4 were 82.2%, 100%, and 0.903; respectively, at a cutoff value of 32.4 (p < 0.001**)**. The sensitivity, specificity, and AUC for PD-1 were 62.2%, 100%, and 0.858; respectively, at a cutoff value of 0.076 (p < 0.001). While those for FOXP3 were 71.1%, 100%, and 0.867; respectively, at a cutoff value of 16.2 (p < 0.001). Regarding microRNAs expression, miR-155 showed 88.9% sensitivity, 96.7% specificity, and 0.934 AUC at a cutoff expression of 0.04 (p < 0.001). The miR-195 showed a 73.3% sensitivity, 60% specificity, and AUC = 0.716 at a cutoff expression of 0.001 (p = 0.001). Meanwhile, MIC-B expression showed a sensitivity of 77.8%, a specificity of 80%, and AUC of 0.811 at a cutoff of 1.17 (p < 0.001**, **Fig. 3).

Combined expression of PDL-1 and CTLA-4 increased the diagnostic significance of BC patients to a sensitivity of 100%, specificity of 100%, and AUC of 1 (**p < 0.001**). Moreover, combined expression of miR-155 and miR-195 achieved a sensitivity of 91.1%, a specificity of 96.7%, and AUC of 0.926 (**p < 0.001**). While adding MIC-B did not add a substantial value to the sensitivity (88.9%), or the specificity (96.7%, Table 4**).**

**Table 4:** Diagnostic value of the assessed markers

	AUC	Cutoff	Sensitivity	Specificity	P value
PDL-1	0.987	1.29	93.3%	100%	p < 0.001
CTLA-4	0.903	32.4	82.2%	100%	p < 0.001
PD-1	0.858	0.076	62.2%	100%	p < 0.001
FOXP3	0.867	16.2	71.1%	100%	p < 0.001
miR-155	0.934	0.04	88.9%	96.7%	p < 0.001
miR-195	0.716	0.001	73.3%	60%	0.001
MIC-B	0.811	0.464	77.8%	80%	p < 0.001
PDL-1 + CTLA-4	1	-	100%	100%	p < 0.001
miR-155 + miR-195	0.926	-	91.1%	96.7%	p < 0.001
miR-155 + MIC-B	0.955	-	88.9%	96.7%	p < 0.001
miR-155 + miR-195 + MIC-B	0.955	-	88.9%	96.7%	p < 0.001

### Correlations among the assessed markers

**Fig. 4:**
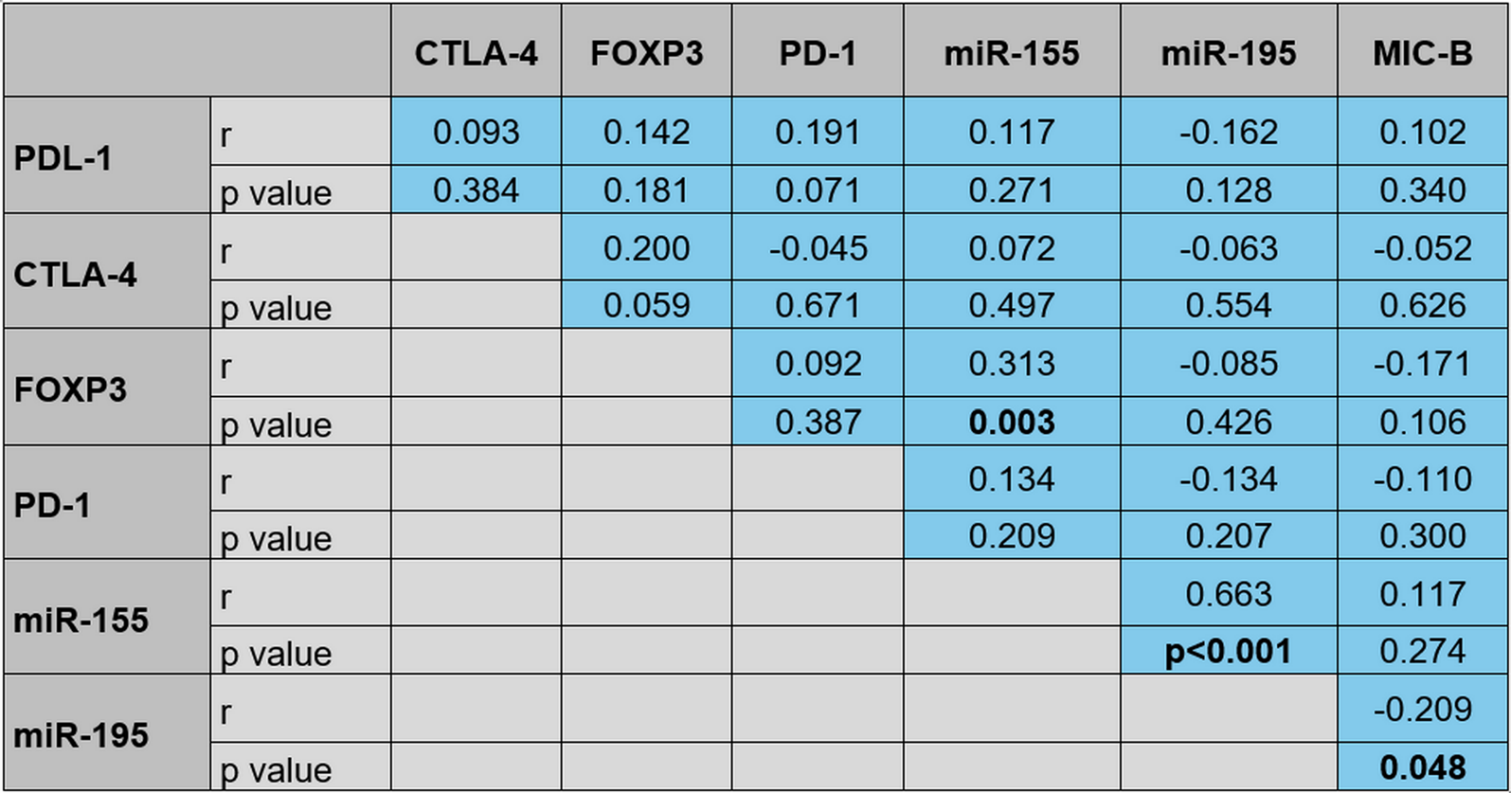
Correlation among PDL-1, CTLA-4, FOXP-3, PD-1, miR-155, miR-195, MIC-B expression levels in breast cancer patients

The BC patients showed a significant inverse correlation between MIC-B expression and miR-195 (r = −0.209**, **p = 0.048**)**. The expression level of miR-155 correlated significantly with FOXP3 expression **(**r = 0.313**, **p = 0.003**)**, and inversely with miR-195 expression (r = −0.663**, **p < 0.001, Fig. 4).

### Regression analysis for breast cancer diagnosis

Univariate regression analysis showed that PDL-1, CTLA-4, PD-1, FOXP3, MIC-B, miR-155, and miR-195expression were significantly associated with BC incidence (p **<** 0.001**, **0.001**, **0.001**, **0.001**,** p < 0.001**, **0.001**,** and 0.001; respectively). Moreover, multivariate regression analysis showed that PDL-1 (OR:13.825, p = 0.004), CTLA-4 (OR: 20.958, p = 0.010), PD-1(OR:10.550, p = 0.044), MIC-B (OR: 17.89, p = 0.003), miR-155 (OR: 211.356, P < 0.001), and miR-195 (OR:0.006, P < 0.001) were considered as independent risk factors for BC (Table 5).

**Table 5:** Multivariate regression analysis for breast cancer diagnosis

	OR	95% C.I for EXP(B)		P value
		Lower	Upper	
Univariate analysis				
PDL-1	27.739	3.622	212.450	**P < 0.001**
CTLA-4	30.318	3.959	232.203	**0.001**
PD-1	14.636	3.289	65.131	**0.001**
FOXP3	13.391	3.009	59.591	**0.001**
MIC-B	22.04	4.542	106.9	**P < 0.001**
miR-155	29	3.787	222.102	**0.001**
miR-195	0.033	0.004	0.253	**0.001**
Multivariate analysis				
PDL-1	13.825	2.266	84.348	**0.004**
CTLA-4	20.958	2.075	211.654	**0.010**
PD-1	10.550	1.068	104.242	**0.044**
FOXP3	4.834	0.724	32.294	0.104
MIC-B	17.89	2.766	115.83	**0.003**
miR-155	211.356	18.971	2354.686	**P < 0.001**
miR-195	0.006	0.001	0.070	**P < 0.001**

Bold variables indicate significant values

## Discussion

Breast cancer is a major health problem in females globally, and its appropriate management depends mainly on the early detection and proper diagnosis of BC patients. The important role of the immune system in cancer pathogenesis and therapy is increasingly recognized and developed. Unlike other tumors, BC is considered a non-immunogenic tumor except for some aggressive subtypes such as triple negative breast cancer (TNBC) (Mittendorf et al. 2007; Adams et al. 2019). It was found that BC cells secret immune inhibitory molecules such as ICIs that attenuate the anti-tumor immune response (Mittendorf et al. 2007). Most of the published articles assessed the role of ICIs in breast tumor tissue. However, in the present study, we aimed at assessing the role and the level of these markers in the PB of the patients as a simple, rapid, and easy tool for the evaluation and early diagnosis of BC patients.

The current research reported a significant increase in the PB of PDL-1, CTLA-4, PD-1, and FOXP3 immunological markers in BC patients compared to the normal control group. These data are consistent with Wang et al., who reported upregulation of PD-1, CTLA-4, and Foxp3 mRNA levels in the PB of cancer patients including BC in relation to normal healthy individuals (Wang et al. 2017). Similarly, Elashi et al. concluded increased expression levels of circulating PDL1 in the PB of BC patients due to aberrant promoter methylation pattern (Syed Khaja et al. 2017). While they found no significant difference detected regarding the expression levels of PD-1 and CTLA-4 in the PB of BC patients compared to healthy control (Elashi et al. 2018). In line with the current results, Erfani et al. (2010), reported that plasma CTLA-4 level is notably increased in the sera of patients with BC compared to the normal reference group. Similarly, Jin et al. (2024), performed a systemic review and concluded that CTLA-4 is notably upregulated in the plasma and tumor tissue of BC patients in comparison to the control groups. In comparison, Bassaro et al., found an increased level of CTLA-4 autoantibodies in the sera of BC patients in relation to the normal group, though it did not reach a significant level (Bassaro et al. 2017). Additionally, Syed et al. also proposed increased FoxP3 + Treg cells in the PB and tumor tissue of BC patients, which might induce an immunosuppressive environment in the BC tissue through increased release of CD39, PD-1 and CTLA-4 molecules. However, they did not find significant differences in PD-1, and CTLA-4 levels in the PB of BC patients and normal controls (Syed Khaja et al. 2017).

Indeed, many studies have confirmed the poor prognostic role of the tumor-infiltrating PDL-1, CTLA-4, PD-1, and FOXP3 immunological markers in BC tissue (Syed Khaja et al. 2017; Tahir et al. 2022; Monneur et al. 2018; Sun et al. 2023), however, the role of the circulating levels of these markers is still a debatable issue. The present data showed that the expression level of FOXP3 increased significantly with tumor grade, while there was no significant association of the PB levels of PDL-1, CTLA-4, and PD-1 and the other clinic-pathological features of the BC patients. In contrast, Syed et al. reported that PB FoxP3 + Treg levels did not differ significantly with disease stage and grade (Syed Khaja et al. 2017). While Jin et al. found that CTLA-4 associated with increased tumor size, cancer cell aggressiveness, advanced tumor stage, and lymph node metastasis (Jin et al. 2024).

Regarding the regulating miRNAs expression, there was a substantial increase in miR-155 level in the PB of BC patients, in comparison to the normal controls. Additionally, the expression level of miR-155 was significantly upregulated in patients with distant tumor metastasis, while there was no significant association with hormonal status or lymph node (LN) metastasis. These results are consistent with many studies reported a substantial upregulation of miR-155 in BC patients in relation with the reference controls (Hassan et al. 2020; Mattiske et al. 2012; Wang et al. 2010; Hou et al. 2016; Anwar et al. 2020; Huang et al. 2018). However, there are many discrepancies regarding the association of miR-155 with the clinical parameters of the patients. Some studies reported increased miR-155 expression with advanced tumor stage and distant metastasis (Hou et al. 2016; Huang et al. 2018; Khalighfard et al. 2018; Swellam et al. 2019), however, other series weakened this association (Anwar et al. 2020; Babaei et al. 2019). Furthermore, in consistent with the present data, Swellam et al. (2019), and Jurkovicova et al. (2017), found no significant association of miR-155 with the hormonal status of the patients, while Wang et al. proposed that serum miR-155 correlated with negative hormonal status (Wang et al. 2010).

There is a conflicting data regarding miR-195 expression in BC patients. The current study showed that the expression level of miR-195 was significantly downregulated in BC patients in comparison to the healthy control group. It was significantly downregulated in patients with positive ER and/or PR expression. Moreover, it was significantly increased in patients with right side BC, in comparison to those with left side BC. These results are consistent with several studies suggesting that miR-195 is significantly downregulated in BC patients, and it acts as a tumor suppressor in BC pathogenesis (Luo et al. 2014; Li et al. 2011; Cecene et al. 2016; Zhao et al. 2014). Similarly, Singh et al., concluded that miR-195 inhibits cancer cell proliferation, migration, aggressiveness, and invasion that make miR-195 a potential targeted therapy for BC patients (Liu et al. 2018). On the other hand, other previously published studies reported overexpression of miR-195 in BC patients (Fan et al. 2018; Heneghan et al. 2010a, 2010b). While McAnena et al., found that miR-195 was significantly downregulated in metastatic BC patients, compared to patients with local disease or healthy controls, however there was no significant differences between normal control and those with BC local disease (McAnena et al. 2019). Moreover, Zhao et al. proposed that there was no significant impact of miR-195 expression on the clinic-pathological chrematistics of the patients including TNM stage, hormonal status, and LN metastasis (Zhao et al. 2014). Therefore, data regarding the role of miR-195 in BC development are still controversial, and more research is required for proper assessment of its diagnostic, prognostic, and predictive value in BC patients.

Growing body of evidence suggested that the proteolytic release of MICA/B from the plasma membrane is a focal mechanism for the evasion of the tumor cells from lysis by NKG2D-expressing immune cells (Baranwal and Mehra 2017; Groh et al. 2002). Therefore, assessment of plasma MICA/B is an important marker for evaluating the tumor immune response of the patients. The present findings revealed a significant decrease in the plasma protein level of soluble MIC-B in BC patients, compared to normal control females. Moreover, it showed a sensitivity of 77.8%, a specificity of 80%, and AUC of 0.811 for the detection of BC patients at a cutoff of 1.17. However, there was no significant association between MIC-B expression and any relevant clinic-pathological features of the assessed patients. These findings are consistent with that reported by Bargostavan et al. (2016), that the serum level of MIC-A and MIC-B were significantly increased in BC patients, where these levels did not correlate with the T allele of the MMP9 (−1562 C/T). Similarly, other series concluded increased serum level of soluble MIC-B in many cancers in relation to the normal controls including gastrointestinal carcinomas, oral squamous cell carcinoma, and lung cancers (Garrido-Tapia et al. 2017; Tamaki et al. 2010; Cascone et al. 2017). On the contrary, Holdenrieder et al. found that the serum level of MIC-B was elevated in cancer patients compared to normal control, but it did not reach a significant level. However, within cancer patients, MIC-B level is significantly associated with advanced cancer stage and distant metastasis (Holdenrieder et al. 2006). These discrepancies in the results could be explained by that the author investigated the serum level of MIC-B in different cancer patients collectively including lung, breast, different gastrointestinal, and gynecological malignancies, which could be differ in BC. This data suggests that the MIC-B serum level may vary according to the malignancy type, which requires further research on different types of cancers. Additionally, Zhao et al., performed a systemic meta-analysis and reported that low serum levels of MICA/B were notably associated with prolonged overall survival in cancer patients (Zhao et al. 2017). Accordingly, Zhang et al. found that increased serum level of soluble MIC-B in cancer patients limited the efficacy of CTLA4 blockade therapy (Zhang et al. 2017). Therefore, administration of a MICB-neutralizing antibody is very necessary for patients receiving CTLA-4 checkpoint inhibitors.

The ROC curve analysis showed that the diagnostic accuracy of miR-155 for BC patients revealed a sensitivity, specificity, and AUC were 88.9%, 96.7%, and 0.934, respectively. In consistent with these data, Hou et al., performed a systematic meta-analysis and suggested that miR-155 could be a potential noninvasive biomarker for cancer detection, it achieved the highest diagnostic power in BC with a sensitivity, specificity, and AUC of 83.8%, 87.5%, and 0.92, respectively (Hou et al. 2016). They added that the diagnostic detection of miR-155 was more precise in Caucasian than in Asian population (Hou et al. 2016). Additionally, Huang et al., found that miR-155 is an accurate diagnostic marker for BC with a sensitivity, specificity, and AUC of 83.3% 80%, 0.817, respectively (Huang et al. 2018). Likewise, other previously published studies proposed that miR-155 could be considered a diagnostic biomarker for BC (Zeng et al. 2014; Liu et al. 2013).

Moreover, the current data revealed that miR-195 showed a 73.3% sensitivity, 60% specificity, and AUC = 0.716 at a cutoff expression of 0.001 for the diagnosis of BC patients. Consistently, Zhao et al. (2014), reported that miR-195 achieved a higher sensitivity (69%) and specificity (89.2%) for the diagnosis of BC patients, in comparison to the carcinoembryonic antigen (CEA, sensitivity: 15.08%) and carbohydrate antigen 15–3 (CA15-3, sensitivity: 21.1%).

Accordingly, combined expression of miR-155 and miR-195 were assessed for the detection of BC. It showed that both miR-155 and miR-195 could be promising diagnostic markers with a sensitivity of 91.1%, a specificity of 96.7%, and AUC of 0.926.

Furthermore, the diagnostic potential of PDL-1, CTLA-4, PD-1, FOXP3, and MIC-B were assessed and revealed that PDL-1, CTLA-4, PD-1, and FOXP3 achieved a specificity of 100% for distinguishing BC patients, at a sensitivity of 93.3%, 82.2%, 62.2%, and 71.1% respectively. Interestingly using both PDL-1 and CTLA-4 scored a 100% sensitivity and 100% specificity for diagnosing BC. Therefore, combined expressions of PDL-1 and CTLA-4 could be promising useful, easy, and reliable markers for BC detection. Both markers are considered strong inhibitory signals for the tumor immune response that cannot be increased consistently and significantly under physiological conditions. As binding of PDL-1 and CTLA-4 with their receptors act as an “off switch” signal for hindering T cells from attacking harmful cells including cancer cells (Tavares et al. 2021). To the best of our knowledge, we couldn’t find other studies that explore the diagnostic significance in the form of sensitivity and specificity of combined PDL-1 and CTLA-4 expressions in cancer patients.

Other supportive data for the diagnostic significance of PDL-1, CTLA-4, PD-1, FOXP3, MIC-B, miR-155, and miR-195in BC patients, univariate regression analysis showed that PDL-1, CTLA-4, PD-1, FOXP3, MIC-B, miR-155, and miR-195expression were significantly associated with BC incidence. Moreover, multivariate regression analysis showed that PDL-1, CTLA-4, PD-1, MIC-B, miR-155, and miR-195were considered as independent risk factors for BC.

miR-155 and miR-195 were proved to be important regulators for PDL-1/PD-1 pathway (Kipkeeva et al. 2022). As miR-155 can induce PD-1/PD-L1 expression directly through binding to the 3′-UTR region (Zheng et al. 2019), or indirectly through upregulating the long non-coding RNAs (MALAT-1) in cancer patients (Atwa et al. 2020). Also, CTLA-4 is a direct target for miR-155 (Jebbawi et al. 2014). On the other hand, miR-195 regulated the tumor immune response by downregulating the expression of PD-1/PD-L1 pathway (He et al. 2018). Consistent to these data, the current study found a significant positive correlation between miR-155 and FOXP3 expression, while an inverse correlation with miR-195 expression. Also, there was a significant inverse correlation between miR-195 and MIC-B expression. In comparison, Tao et al., reported a negative correlation between the expression level of miR-195 and PD-L1, PD-1, CD80, and CTLA-4 in prostate cancer (Tao et al. 2018).

In conclusion, the current study provided evidence that the PB levels of PDL-1, CTLA-4, PD-1, FOXP3, MIC-B, miR-155, and miR-195 could be used as promising diagnostic markers for BC patients. The Combined expression of PDL-1 and CTLA-4 increased the diagnostic significance of BC patients to a sensitivity of 100% and a specificity of 100%. Also, the combined expression of both miR-155 and miR-195 achieved a sensitivity of 91.1%, a specificity of 96.7%, and an AUC of 0.926. Moreover, PDL-1, CTLA-4, PD-1, MIC-B, miR-155, and miR-195 were considered as independent risk factors for BC. Therefore, further research is required to assess the exact role of each marker in BC pathogenesis and development. This will provide new insight into personalized immune therapy for BC patients.

Regarding the study’s limitations, the tested markers were assessed in a relatively small number of patients, which required to be validated on a larger number of patients with different pathological BC subtypes. In addition, the PB results should be correlated with the corresponding BC tissue samples for a better understanding of the interplay of these markers with the tumor pathogenesis and the reflecting clinical outcomes of the patients.

Many ICIs were approved by the FDA for the treatment of advanced-stage BC patients positive for PDL-1 (Rakha et al. 2022). However, unlike to other tumors, PDL-1 assessment is not performed in routine practice for BC patients, except in limited centers. Therefore, it is recommended for considering PDL-1, CTLA-4, PD-1, FOXP3, MIC-B, miR-155, and miR-195 in the diagnosis and evaluation of the clinical and immunological status of BC patients, who are eligible for targeted immunotherapy. Moreover, miR-155 and miR-195 could be potential successful targets for cancer therapy.

## Data Availability

No datasets were generated or analysed during the current study.
